# Ignored sediment fungal populations in water supply reservoirs are revealed by quantitative PCR and 454 pyrosequencing

**DOI:** 10.1186/s12866-015-0379-7

**Published:** 2015-02-22

**Authors:** Haihan Zhang, Tinglin Huang, Shengnan Chen

**Affiliations:** School of Environmental and Municipal Engineering, Xi’an University of Architecture and Technology, Xi’an, 710055 Shaanxi Province P. R. China

**Keywords:** Fungal population, 454 GS FLX pyrosequencing, 18S rRNA gene, *Glomus* sp, Quantitative PCR

## Abstract

**Background:**

The sediment hosts a variety of fungal species in water supply reservoirs; however, the taxonomically and functionally diverse fungal populations have remained vastly unexplored. Here, quantitative PCR (qPCR) and recently developed high-throughput 454 GS FLX pyrosequencing were combined to investigate the abundance and diversity of sediment fungal communities in three water supply reservoirs.

**Results:**

These results revealed 1991, 2473, and 2610 copies of the 18S rRNA gene in the sediments from the ZC, SBY, and JP reservoirs, respectively. The fungal abundance in JP reservoir was 1.31 times higher than that of the ZC reservoir. In general, 43123 reads were recovered, corresponding to 945 distinct molecular operational taxonomic units (OTUs, 97% similarity cut-off level). The majority of the fungal nuclear ribosomal internal transcribed spacer (ITS) region sequences were affiliated with Ascomycota, Chytridiomycota, Basidiomycota, Glomeromycota, and Mucoromycotina. The highest *Chao* 1 index (962) was observed in the JP reservoir, and this value was 5.66 times greater than that of the SBY reservoir. Heat map analysis showed that *Rhizophydium* (relative frequency 30.98%), *Placidium* (20.20%), *Apophysomyces* (8.43%), *Allomyces* (6.26%), and *Rhodotorula* (6.01%) were the dominant genera in the JP reservoir, while *Elaphomyces* (20.0%) was the dominant genus in the ZC reservoir and *Rhizophydium* (30.98%) and *Mattirolomyces* (39.40%) were the most abundant in the JP and SBY reservoirs. *Glomus* sp. was only found in the JP reservoir. Furthermore, the larger proportions of “unassigned fungi” call for crafting International Nucleotide Sequence Database. Principle component analysis (PCA) and network analysis also suggested that tremendously diverse functional fungal populations were resident in the sediments of the three water supply reservoirs.

**Conclusions:**

Thus, the results of this research suggest that the combination of high-throughput Roche 454 GS FLX pyrosequencing and qPCR is successfully employed to decrypt reservoir sediment fungal communities. Diverse fungi occur widely in the sediments of water supply reservoirs. These findings will undoubtedly broaden our understanding of reservoir sediment fungal species harbored in this freshwater stressful environmental condition. Future research should be conducted to determine the potential for fungi to degrade complex pollutants and their secondary metabolites related to the water quality.

## Background

Aquatic sediment hosts diverse microbial communities that are the main drivers of nutrient cycles and energy fluxes [1-3]. Over the past few decades, bacterial and archaeal communities from the sediment of various aquatic environmental systems have been widely examined [4-8]. Sediment carbon monoxide-oxidizing bacteria [2], methanobacteria [3], denitrifying bacteria [1], and sulfate-reducing bacterial community compositions [9] clearly regulate the exchanges and transformation of carbon (C), nitrogen (N), and sulfur (S) at the water-sediment interface of eutrophic lake [2], constructed wetland [10], and acidic mine-draining river [9]. In contrast, sediment fungal community structure and diversity are not well examined although fungal species perform important services involvement in organic matter decomposition and food web [11].

Reservoirs provide water sources for several beneficial purposes, including agricultural irrigation, industrial cooling processes, and urban municipal water utilization [12]. To ensure the security of urban water supply, determining of the harmful cyanobacterial toxins [13], antibiotic resistance genes [14], and endogenous pollutants (e.g., nitrogen, phosphorus, iron, and manganese) released from sediments [15] of water supply reservoirs has been routinely performed. From an aquatic ecological point of view, more comprehensive exploration of the microbial diversity in sediment will improve our understanding of the major global biochemical processes in reservoirs [3]. Unfortunately, limited publications have revealed sediment microbial compositions [4,5], and even fewer have described fungal abundance and communities [11].

Sediment fungi represent a significant component of the benthic microbial biomass in reservoirs, and these organisms are a vital biological force in regulating water quality through decomposition of organically bound C and N deposited on the bottom [15-17]. Our previous studies of water supply reservoir sediments demonstrated that sediment fungal communities successfully utilized carbohydrates, phenolic compounds, and carboxylic acids as carbon sources [16,17]. Furthermore, nested polymerase chain reaction-denaturing gradient gel electrophoresis (PCR-DGGE) profiles suggested that the sediment fungal community was fairly complex; however, more specific taxonomically defined fungal populations have not yet been thoroughly explored due to technological limitations such as only predominant species are displayed in the DGGE gel fingerprints and the co-migration of PCR fragments with different sequences [17].

With notable advances in molecular and bioinformatics technologies, several useful methods such as catalyzed reporter deposition fluorescence in situ hybridization (CARD-FISH), clone sequencing, and functional gene arrays (FGAs, GeoChip) have been widely employed for tracking sediment microbial diversity, and the application of high-throughput sequencing techniques (HTST) has provided insight into reservoir sediment microbial communities [4,5,18]. Röske et al. [4,5] used CARD-FISH and 454 GS FLX pyrosequencing to investigate sediment bacterial and archaeal communities from a mesotrophic drinking water reservoir located in Saxony, Germany. Recently, Huerta et al. [14] also determined the sediment bacterial communities in three water supply reservoirs located near Barcelona, Spain using Roche 454 GS FLX pyrosequencing.

The present study described here will help to close this enormous “fungi gap” in our understanding of sediment microbial communities via using Roche 454 GS FLX pyrosequencing to provide detailed genetic fingerprints of sediment fungal community diversity in water supply reservoirs with different eutrophication level. The main objective of the present study was to determine the abundance and diversity of fungal community in the surface sediments from three water supply reservoirs (named JP, SBY, and ZC reservoirs) with different eutrophication levels. To this end, we (1) utilized quantitative PCR (qPCR) to examine the 18S rRNA gene copy numbers and (2) used 454 GS FLX pyrosequencing to determine the taxonomic diversity and composition of the fungal community compositions in the sediments of the JP, SBY, and ZC reservoirs. The results from this work can give us greater insight into the aquatic fungal community diversity in beneath reservoir sediment exposed to various degrees of eutrophication.

## Results

### Sediment fungal abundance and diversity

The abundance of sediment fungal communities from JP, SBY and ZC reservoirs were determined by quantitative PCR. The abundances of fungal 18S rRNA gene sequences in the three reservoir sediment samples were presented in Table 1. The fungal 18S rRNA genes ranged from 2610 ± 89 copies [g dry sediment]^−1^ to 1991 ± 58 copies [g dry sediment]^−1^ in the sediment samples. The fungal abundance in JP reservoir was 1.31 times higher than that of the ZC reservoir (*P* < 0.01).

**Table 1:** **Quantitative PCR and fungal population diversity index based on the 454 pyrosequencing data from sediments samples from the JP, SBY, and ZC reservoirs**

**Reservoirs**	**Fungal 18S rRNA gene copies per gram sediment** *	**Abundance-based coverage estimators (ace)**	**Shannon’s diversity (** ***H*** **)**	**Simpson diversity (** ***D*** **)**	***Chao*** **1 diversity**	**Coverage**
ZC reservoir	1991 ± 58 B	307	1.25	0.50	328	0.975
SBY reservoir	2473 ± 102AB	190	0.96	0.57	170	0.994
JP reservoir	2610 ± 89A	967	4.52	0.05	962	0.993

*The data shown are the means (*n* = 3). The same letter (A or B) indicates no significant difference by Tukey-Kramer HSD (*P* < 0.01).

**Figure 1:**
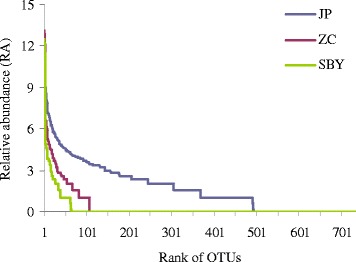
**Rank-abundance curve based on the fungal nuclear ribosomal internal transcribed spacer (ITS).** OTUs at a dissimilarity level of 3% in the sediments of the ZC, JP, and SBY reservoirs.

**Figure 2:**
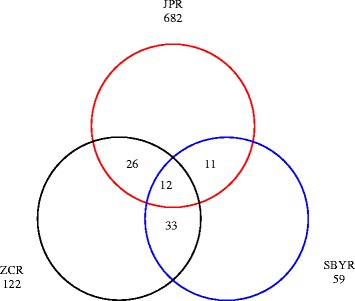
**The Venn diagram depicts distinct and uniform fungal nuclear ribosomal internal transcribed spacer (ITS).** OTUs at the 97% similarity cut-off level for the sediment fungal populations from the ZC, SBY, and JP reservoirs (ZCR, SBYR, and JPR, respectively).

The present work validates the powerful and effectiveness of 454 pyrosequencing method for the survey of sediment fungal composition diversity. It revealed a high diversity of fungal compositions with a total of 43123 raw ITS sequences (18425, 11554, and 13144 for ZC, SBY and JP, respectively) with an average length about 650 bp obtained from the three reservoirs sediment samples. After filtering, 30880 high-quality sequences were selected for the data analysis. As shown in Figure 1, the rank-abundance curves indicated that the JP reservoir had a highest species richness and evenness, whereas the SBY reservoir showed the lowest species richness (shortest curve) and also the lowest species evenness (lowest curve). To determine fungal diversity, OTUs were identified based on the fungal ITS sequence with a dissimilarity level of 3%. In total, 945 OTUs were detected. As shown in Table 1, the JP reservoir sample had the highest richness (ace = 967, *Chao* 1 = 962), while the sample from SBY had the lowest richness (ace = 190, *Chao* 1 = 170). Furthermore, the JP reservoir sample exhibited the highest diversity (*H* = 4.52) of the three samples, and this diversity was 3.6 times higher than that of the ZC reservoir (*H* = 1.25). The SBY reservoir exhibited the lowest diversity (*H* = 0.96). The lowest Simpson diversity (*D*) was observed in the JP reservoir (Table 1). In addition, the JP, ZC, and SBY reservoirs had 682, 122, and 59 OTUs in common, respectively. The OTUs shared among the three reservoirs were depicted using a mothur Venn diagram, and 12 OTUs were shared with three reservoirs (Figure 2).

### Sediment fungal community composition

As shown in Table 2, the taxa detected in our sampling cover a wide variety of organisms from the five main phyla and include Ascomycota, Basidiomycota, Chytridiomycota, Glomeromycota, Mucoromycotina, and unassigned. In three reservoirs, the highest phylum is Chytridiomycota (12.71% in JP, 9.43% in SBY, 2% in ZC), and the lowest phylum is Glomeromycota (1.50% in JP, 0.83% in ZC, 0.0% in SBY). Unfortunately, at the phylum level, 66.38% (JP), 85.41% (ZC), and 79.25% (SBY) of the sequences could be identified as “unassigned fungi”. Clearly sediment harbors diverse ecosystems with a plethora of ignored fungal species. These larger proportions of “unassigned fungi” call for curatted International Nucleotide Sequence Database (INSD). Dominant classes among the Ascomycota were the Dothideomycetes, Eurotiomycetes, Pezizomycetes, Saccharomycetes and Sordariomycetes. The fungal communities were dominated by OTUs belonging to the Chytridiomycota (33.90% of OTUs). Dominant Chytridiomycota groups included the Blastocladiomycetes, Chytridiomycetes and Monoblepharidomycetes. According to the Parsimony test, no significant difference existed between the ZC and SBY reservoirs for Ascomycota and Chytridiomycota (*P* > 0.05); however, the JP reservoir was significantly different from the ZC and SBY reservoirs at the phylum level (*P* < 0.01).

**Table 2:** **Parsimony test analysis for the taxonomic distribution of the sediment fungal community based on 454 pyrosequencing data for the ZC, SBY, and JP reservoirs**

**Fungal phylum, and class** ^**a**^	**ZC** ***vs.*** **SBY (** ***P*** **-value)**	**JP** ***vs.*** **ZC (** ***P*** **-value)**	**JP** ***vs.*** **SBY (** ***P*** **-value)**
**Ascomycota**	NS	<0.01	0.01
Dothideomycetes	NS	0.01	0.01
Eurotiomycetes	NS	0.01	0.01
Pezizomycetes	0.01	NS	0.01
Saccharomycetes	NS	0.01	0.01
Sordariomycetes	NS	<0.05	0.05
**Basidiomycota**	0.01	0.01	0.01
Agaricomycetes	0.01	NS	0.01
Agaricostilbomycetes	NS	0.01	0.01
Microbotryomycetes	NS	0.01	0.01
Tremellomycetes	NS	NS	NS
**Chytridiomycota**	NS	0.01	0.01
Blastocladiomycetes	NS	0.01	0.01
Chytridiomycetes	NS	0.01	0.01
Monoblepharidomycetes	NS	0.01	0.01
**Glomeromycota**	0.01	0.01	0.01
Glomeromycetes	0.01	0.01	0.01
**Mucoromycotina**	NS	0.01	0.01
Mortierellales^b^	NS	0.01	0.01
Mucorales	NS	0.01	0.01
**Unassigned fungi**	0.01	0.01	0.01

^a^NCBI taxonomy. ^b^Order. NS represents not significant, *P* > 0.05.

**Figure 3:**
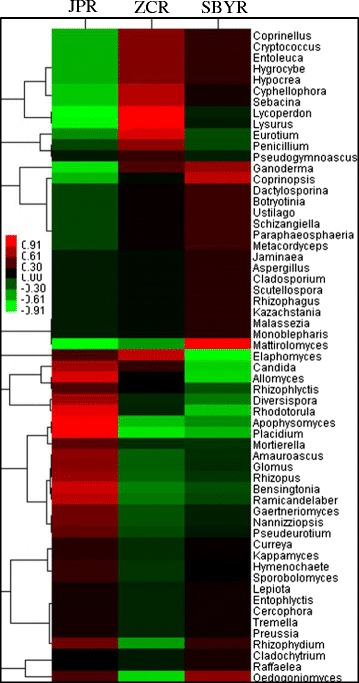
**Heat map diagram depicts sediment fungal populations**. The colors (from −0.91 to 0.91) show the relative abundances of fungal nuclear ribosomal internal transcribed spacer (ITS) OTUs at the 97% similarity cut-off level. ZC, SBY, and JP reservoirs (ZCR, SBYR, and JPR, respectively).

More specifically, *Rhizophydium* (relative frequency 30.98%), *Placidium* (20.20%), *Apophysomyces* (8.43%), *Allomyces* (6.26%), and *Rhodotorula* (6.01%) were the dominant genera in the JP reservoir, while *Elaphomyces* (20.00%) and *Rhizophydium* (13.84%) dominated in the ZC reservoir and *Rhizophydium* (77.78%) and *Oedogoniomyces* (16.67%) dominated in the SBY reservoir. *Glomus* sp. was only found in the JP reservoir. Based upon the relative percentages of the main 59 fungal types, we utilized heat map diagram colors to represent the relative percentages of the fungal classes within each reservoir (Figure 3).

**Figure 4:**
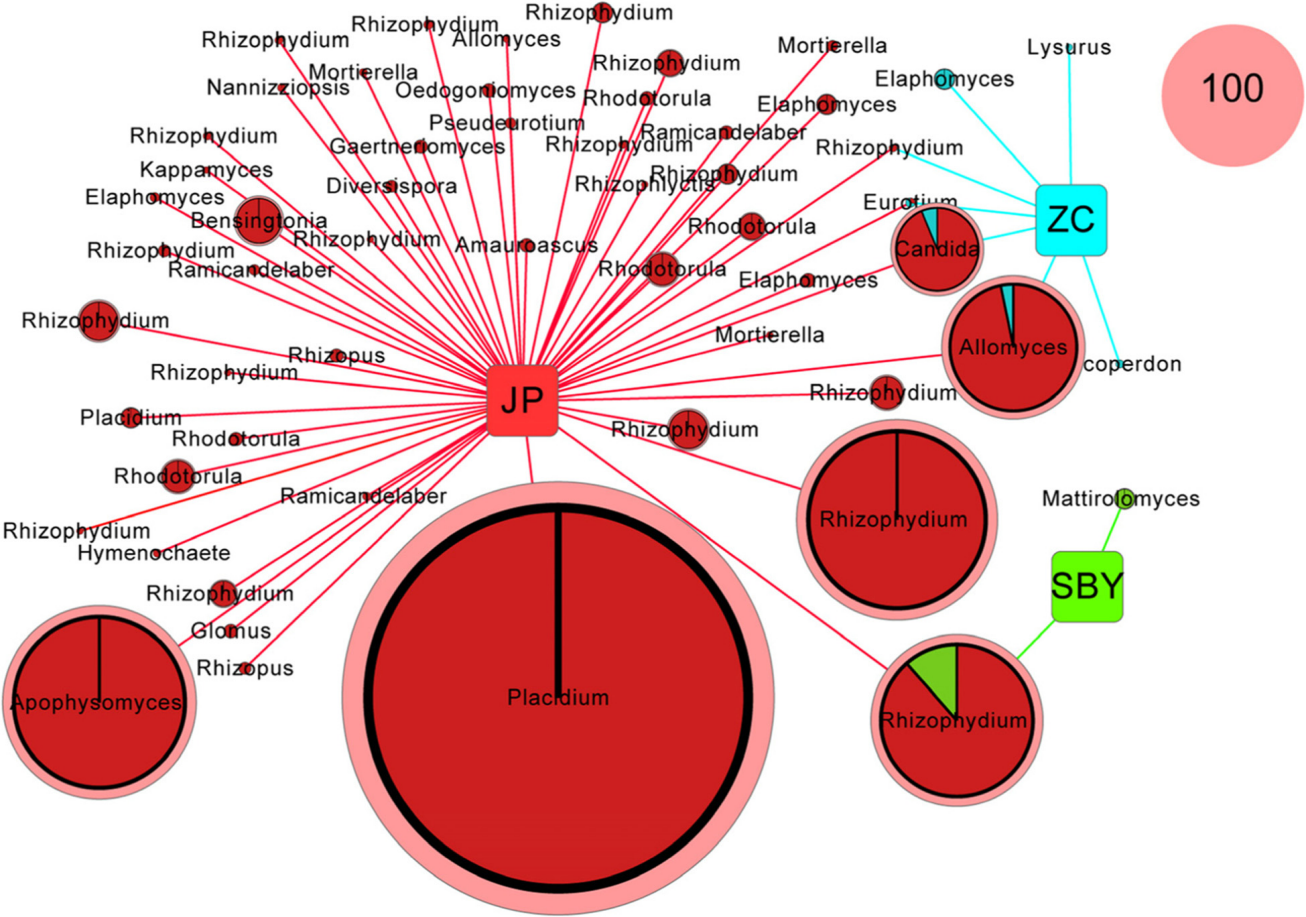
**Cytoscape network clustering diagram.** The figure shows the dominant fungal nuclear ribosomal internal transcribed spacer (ITS) OTUs at the 97% similarity cut-off level from ZC (azure), SBY (green), and JP (red) reservoirs. Node sizes represent relative abundance. The standard node indicates 100 reads.

**Figure 5:**
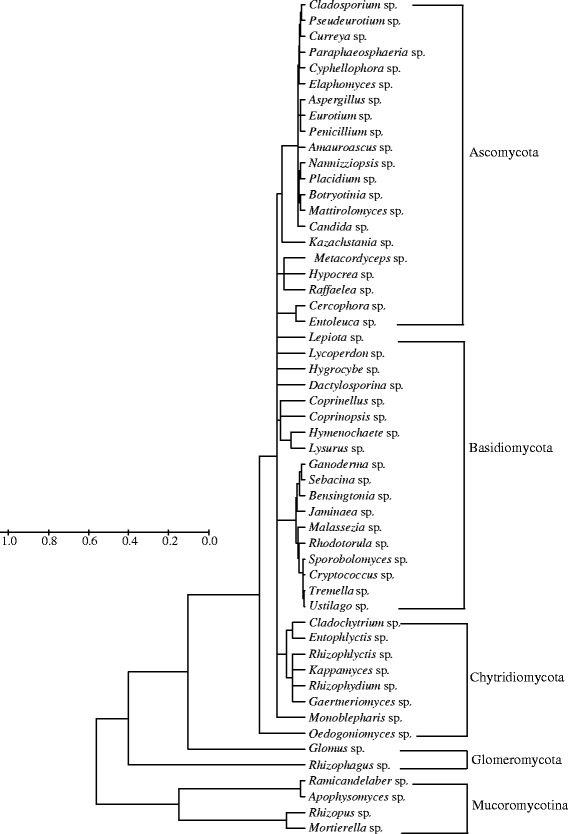
**Phylogenetic relationships among the 53 OTUs with internal transcribed spacer (ITS) sequences of known fungi based on the neighbor-joining analysis.** Bootstrap values were based on 1000 replications.

**Figure 6:**
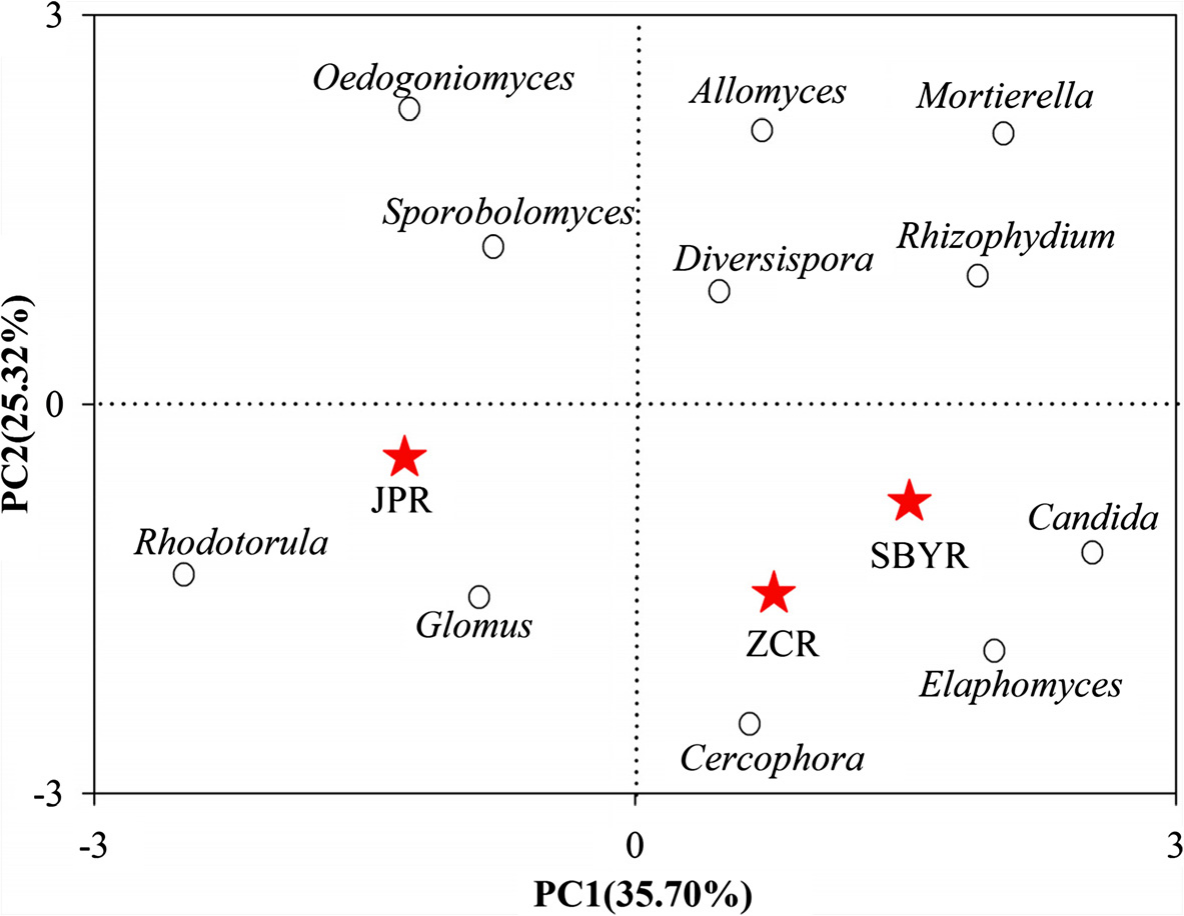
**Principle component analysis (PCA) of sediment fungal populations.** Sampling sites from ZC, SBY, and JP reservoirs (ZCR, SBYR, and JPR, respectively). PC1 and PC2 explained the total variance for 35.70% and 25.32%, respectively.

To better understand the distinct communities of the three reservoirs, we employed diagram clustering network analysis. These findings showed that *Placidium* was the most ubiquitous fungal genus and was dominant in the JP reservoir (Figure 4). Thus, these two types of dendograms enabled us to visualize the entire dataset. Furthermore, phylogenetic tree was shown in Figure 5, the abundant genuses were belonged to Ascomycota, Basidiomycota, Chytridiomycota, Glomeromycota and Mucoromycotina. Principle component analyses (PCA) also revealed that the sediment fungal community structure varied significantly among the reservoirs. Principle component 1 (PC1) and Principle component 2 (PC2) explain 35.70% and 25.32% of total variance, respectively. JPR was located in the third quadrant, and ZCR and SBYR were located in forth quadrant. *Lysurus* sp. and *Lycoperdon* sp. were presented in ZCR, *Mattirolomyces* sp. was in SBYR, *Placidium* sp., *Rhodotorula* sp., *Glomus* sp. were abundant in JPR (Figures 3, 4, 6).

## Discussion

Compared with other freshwater bodies such as lakes, rivers, streams, and springs, reservoirs might be less explored. There is plenty of literature showing that fungi contribute more to leaf breakdown than bacteria in other aquatic environments [19]. The environmental conditions of oligotrophic freshwater supply reservoir sediments are unique with temperature about 8-10°C, and during thermal stratification, the reservoir sediments are exposed to anaerobic conditions [11,20]. Evidence has suggested that these allotrophic environments harbor great bacterial structures [5]; however, enumeration of reservoir sediment fungal diversity is still lacking. Thus, additional works on the fungal community in the previously undocumented water supply reservoir sediments are required. Importantly, in reservoir ecosystems, microbial species, such as bacteria and archaea, are important for energy flow [16,17]; however, microbial species are not limited to these species, and fungi are prevalent in freshwater reservoirs and play major roles in regulating the flow of organic carbon and nitrogen [15,21]. Furthermore, the water quality and sediment physicochemical properties also shape the fungal community in the sediment and in water overlying the sediment [4,5]. Freshwater reservoir sediment fungi are only beginning to be revealed in recent years [16,17,19]. To close this gap, this study presents the more comprehensive dataset on fungal community composition from the sediment of water supply reservoirs.

Over the past several decades, culture-dependent fungal analysis methods, such as the horse hair baiting method [22], bait method [23], Czapek-Dox agar, Sabouraud dextrose media [24], and community-level physiological profiles (CLPPs) [16], have been used to examine the occurrence of fungi in sediments from rivers and reservoirs. The fungal species in reservoir sediments are mainly organisms with unknown physiological activity [22,23]. Our previous study using BIOLOG micro-FF plate to investigate sediment fungal community functional diversity from the SBY and TY reservoirs suggested that tremendous fungal diversity with respect to carbon utilization profiles was present in sediments of the SBY and TY reservoirs [16]. These studies reported a remarkable physiological metabolic potential of fungi that inhabit these sediments, although the phylogenetic diversity of these fungi are only beginning to be revealed.

Recently, much attention has been focused on HTST platforms including 454 GS Junior (Roche), Ion Torrent PGM (Life Technologies), and MiSeq (Illumina) [18]. The 454 GS FLX pyrosequencing techniques are quite powerful and are increasingly explored to evaluate the aquatic microbial communities [5,18,25]. Our results suggest that sediment fungi are important benthic organisms that have previously been ignored because of the constraints of the available test methods. Culture-dependant techniques, such as plate-pouring and Biolog methods, have incompletely assessed the fungal landscape in sediments [16]. To this end, we utilized a next-generation, high-throughput 454 pyrosequencing approach to overcome these difficulties and reveal the sediment fungal community diversity from three different reservoirs in China. Furthermore, the abundance of fungal species was also examined based on quantitation of fungal 18S rRNA genes using a qPCR assay.

The sediment fungal community in reservoirs was both taxonomically diverse and OTU-rich. Our results revealed the abundance of previously unknown fungi in the sediments. In addition, different fungal abundance was observed among three reservoirs might be duo to different reservoir had distinct water quality and environmental conditions [19]. The 18S rRNA gene copy number obtained from this study was lower than that of the arable soils [26]. Rousk et al. [26] examined soils collected across a long-term liming experiment, and revealed 2000–8000 18S rRNA gene copy numbers. Next-generation sequencing allows a deeper insight into sediment fungal communities in the reservoirs. The result of PCA agrees with another study that revealed distinct fungal compositions in samples collected from five different trophic status reservoirs [27]. The fungal communities from three reservoir sediments were characterized by specific and distinct structures. These oligotrophic water source reservoirs harbor a high diversity of largely unknown fungal species. Phylogenetic diversity of the fungal community is separated among the three reservoir sediments. Our results shows Chytridiomycota dominated over Basidiomycota and Ascomycota with respect to abundance in these sediments. In the present study, results revealed that *Rhizophydium* was the dominant genera in all of the reservoirs. *Rhizophydium* is one of two genera in the Chytridiales with more than 220 described species. This result may indicate that the presence of these fungal taxa play an important role in nutrient recycling in the sediment ecosystem, meanwhile, the sediment fungal species were also mainly depended on the degree of water contaminations [15,16]. However, as shown in our previous studies, the water quality of JP reservoir was best than SBY reservoir and ZC reservoir [11,20]. It is suggested that the diversity of aquatic hyphomycetes is highest in relatively non-polluted reservoir. Similarly, Sridhar et al. [28] also found lower fungal diversity in the more nutrient-enriched stream. The most possible explain for this phenomenon was high levels of toxic organic and inorganic micropollutants typically associated with eutrophication might be decreased fungal community diversity [19].

In this work, the JP reservoir was dominated by *Rhizophydium*, while the ZC reservoir was dominated by *Elaphomyces* and the SBY reservoir was dominated by *Rhizophydium* as well as *Oedogoniomyces*. The distinction in fungal abundance and community structure in this region may be related to sediment physicochemical characteristics [8,29]. *Rhodotorula* sp. has been previously isolated from deep-sea sediments in the northwest Pacific Ocean [30]. The dominance of *Oedogoniomyces* was also found in high-elevation soils [31]. Our results are in agreement with the results of other study that examined reservoir samples [27]. For example, Ranković used culture-dependent methods to investigate the presence of fungi in Serbian reservoirs and found that *Penicillium, Rhizopus*, and *Rhizophydium* were popular [27].

Furthermore, we unexpectedly found that *Glomus* sp. was abundant in the JP reservoir sediment samples, although *Glomus* sp. is a typical arbuscular mycorrhizal fungi (AMF), which typically penetrates the cortical cells of vascular plant roots [32]. As no plants were growing at the bottom of this deep reservoir, the most likely reason for this phenomenon is that various vascular plant species are growing on the mountains around this valley reservoir, especially in the water level-fluctuation zone [33]. Previously, we found that *Setaria viridis* was the dominant plant grown in the middle of water level fluctuation zone in the JP reservoir, and *Glomus* sp. consistently colonizes the root of *S. viridis* [33]. Storm run-off-induced water level changes may carry AMF spores, such as *Glomus* sp., from rehizosphere soils along with the roots into the reservoir, and then this matter settles down into the sediments. This result was consisted with Anderson et al. [34] suggested that *Glomus* sp. had been identified from lake sediment cores from Gould Pond and Upper South Branch Pond, Maine, USA. As far as we know, this is the first report of *Glomus* sp. harbored in deep reservoir sediment environmental conditions.

Chytridiomycota dominated the fungal biodiversity in the SBY and JP sediments. A similar study performed by Kagami et al. [35], which employed DGGE and sequence analysis, demonstrated that a large proportion of the sequences belonged to chytrids in Inba Lake in Japan. Microscopic observations revealed that chytrids infect various algal species, such as *Aulacoseira granulata* and *A. ambigua* [35]. This observation is also in agreement with other work performed on high-elevation soils [31]. In the United States, for example, high Chytridiomycota abundance was also detected in the high-elevation soils undergoing snowmelt. This environmental ecosystem has no plants and fewer carbon source inputs than the reservoir system. Freeman et al. [31] suggested that chytrids can utilize algae and cyanobacteria as carbon sources for survival. In agreement, various algae and cyanobacteria live in the reservoir water and sediments [11,20]. The water content of sediment is high. Our previous study found that diatom species are very popular in reservoir sediments. Bertrand et al. [36] revealed parasitic and saprotrophic chytrids are a significant component of freshwater fungi that inhabit the pelagic algal. *Rhizophydium* was dominated in JP and SBY reservoirs. Likewise, Fernández et al. [37] also found that the parasitism by *Rhizophydium couchii* played a vital role in the dynamics of the *Closterium aciculare* community in a eutrophic-hypertrophic reservoir from Argentina. It might be that freshwater reservoir sediment conditions, therefore, was very suitable for chytrids survival and growth duo to direct parasitism in freshwater algal cell.

## Conclusions

In conclusion, aquatic fungi play a major role in biogeochemical processes in freshwater reservoir. Little is known, however, the abundance and diversity of sediment fungal community in water supply reservoir. This study is one of the few studies that combined qPCR with 454 high-throughput pyrosequencing-based evaluation of the fungal community diversity in water supply reservoir sediments. We successfully described highly diverse fungal communities in the sediment of three different water supply reservoirs. Our results showed that the 18S rRNA gene was found in abundance in the sediments from the ZC, SBY, and JP water supply reservoirs. The fungal abundance in JP reservoir was 2.19 times higher than that of the ZC reservoir. The detected OTUs were broadly distributed across *Rhizophydium*, *Elaphomyces, Mattirolomyces, Placidium*, *Apophysomyces*, *Allomyces*, and *Rhodotorula* genera. Likewise, our data also show that the sediment fungal communities are significant distinct among the three water supply reservoirs. *Rhizophydium* (relative frequency 30.98%), *Placidium* (20.20%), *Apophysomyces* (8.43%), *Allomyces* (6.26%), and *Rhodotorula* (6.01%) were the dominant genera in the JP reservoir, while *Elaphomyces* (20.00%) and *Rhizophydium* (13.84%) dominated in the ZC reservoir and *Rhizophydium* (77.78%) and *Oedogoniomyces* (16.67%) dominated in the SBY reservoir. *Glomus* sp. was only found in the JP reservoir. Sediment endogenous pollutants releasing and organic carbon sequestration are mainly mediated by the sediment fungi. Understanding the diversity of sediment fungal community is essential for predicting the further ecological function. In order to shed additional light on these issues, we will determine the effects of water lifting and aeration on water and sediment fungal communities and function using metagenomic library-based combined with protein two-dimensional electrophoresis and stable isotope probing [38,39] techniques in the future.

## Methods

### Sampling sites description and DNA extraction

This study was conducted on three different water supply reservoirs (named JP, SBY, and ZC reservoirs) in China. Our research group members typically monitor the water quality of these seasonally stratified reservoirs, which are oligotrophic, although ZC reservoir often exhibits moderate eutrophication development trend [12,20]. As shown in our previous reports [12,20], the water quality of the ZC reservoir is worse than the JP and SBY reservoirs due to the presence of intense fish farming ten years ago.

The JP reservoir, a potable water source reservoir, is located in Zhouzhi County (34°07′N, 108°20′E), Shaanxi Province, northwestern China. The annual average rainfall is approximately 900 mm. This reservoir was built in 1996 and includes 1481 km^2^ of watershed and 4.55 km^2^ of water surface. The maximum depth is 90 m, and the daily water supply ability for Xi’an city is about 8.0 × 10^5^ m^3^ [12].

The SBY reservoir, which was built in 1976, is situated in Chang’an district (34°00′N, 108°95′E) and is 35 km from Xi’an City, Shaanxi Province, northwestern China. The maximum and minimum water levels are 731 m and 675 m above sea level, respectively. The maximum depth is about 50 m. The daily water supply for Xi’an City is about 4.0 × 10^5^ m^3^. The total storage capacity is 2.81 × 10^8^ m^3^. The height of the dam is 82.5 m, and the length of this dam is 285 m [20].

The ZC reservoir, which was built before 1985, is located in Zaozhuang City (34°56′N, 117°40′E), Shandong Province, eastern China. The maximum depth is about 15–18 m, with an average depth of about 13 m, and the area of the water surface is 6.46 km^2^ [17]. This reservoir serves as an important backup water source for Zao Zhuang City municipal water utilization during the dry season.

In summer and autumn, stable thermal stratification is seasonally formed in these three water supply reservoirs [12,20]. During the sampling periods, the dissolved oxygen concentration of sediment-overlying water is about 0.2 mg/L, the bottom sediment was experiencing complete anaerobic conditions with approximately 8 ~ 10°C [12]. As previously described [17], the surface sediment cores (~30 cm long) were sampled using a sterilized Peterson sampler. Sediment samples were collected randomly from three spatially separated sites in each reservoir with a minimum of 90–100 m interval. The upper 5 cm (0-5 cm depth) of the surface sediments from the triplicate samples in each reservoir was sliced and mixed, placed into sterile polyethylene bags (Corning, Biotechnology Co., Ltd, Shanghai, China) and then maintained at 8°C in a cooler (SK-01A, XBY technology Co., Ltd, Beijing, China) until transfer into the laboratory of School of Environmental and Municipal Engineering, Xi’an University of Architecture and Technology (SEME-XAUAT) within 24 hours after fieldwork.

Samples were sieved through a sterile stainless steel 2 mm sieve and then frozen at −20°C using for DNA extraction. Total microbial DNA was extracted from 0.5 g sediment (wet weight) as reported previously [17] using soil fast DNA® extraction kit (Omega Bio-Tek, Norcross, GA USA) according to the manufacturer’s protocol. The extracted DNA was purified using DNA purification kit (DP209, Tiangen Biotechnology Co., Ltd, Beijing, China), and the DNA concentration and quality (OD_260_/OD_280_ ratio) were determined using ND 2000 Nanodrop (Thermo Scientific, Waltham, MA, USA), and checked on 0.8% agarose gel (Amresco, OH, USA), visualized by ethidium bromide (0.5 mg/L, Sigma, USA) staining and UV illumination (Bio-Rad, Gel Doc™ XR^+^, USA). The purified DNA was stored at −20°C until quantitative PCR and 454 pyrosequencing analysis.

### Quantitative PCR analysis

To examine the relative abundance of sediment fungi in three reservoirs, we used real-time quantitative PCR (qPCR) methods as described by Rousk et al. [26] and Dollive et al. [40] with minor modifications. In this work, an assay was used determining the 18S rRNA gene. The qPCR reaction was performed in a volume of 25 μl with 2× SYBR Green qPCR Master Mix (12.5 μl, TaKaRa, Japan), 1 μl of each primer (10 μM), ddH_2_O (9.5 μl), and 2 μl of DNA template (45 ng/μl). The following primers were used in these studies: Fung (5′-ATTCCCCGTTACCCGTTG-3′) and NS1 (5′-GTAGTCATATGCTTGTCTC-3′). The qPCR was performed in a IQ 5 thermal cycler (Bio-Rad, Hercules, CA, USA) using the following amplification protocol: 95°C for 2 min followed by 40 cycles of 95°C for 10s, 60°C for 40 s, and 72°C for 30s. Samples were then maintained at 4°C, and checked on the 0.8% agarose gels (Amresco, OH, USA) with DL 2000 DNA Marker (Kangwei Biotech Co., Ltd, Beijing, China), stained with ethidium bromide (5 mg/L, Sigma, USA). The resultant qPCR product of 337 bp was obtained.

The standard curve was constructed using genomic DNA containing a full-length copy of the *Saccharomyce cerevisiae* 18S rRNA gene. Ten-fold serial dilutions from 10^−1^ to 10^−7^ were used to generate the standard curve [26]. The calculated DNA melting curve ranged from 80°C to 88°C with 0.5°C increments. The average amplification efficiency (AAE) was 91.03%, and amplification resulted in a good linear relationship (*R*^2^ = 0.998). Based on the number of gene copies from the standard curve, the tested fungal 18S rRNA gene copy numbers were calculated according to cycle threshold (*C*_t_) data [26]. All qPCR reactions were repeated in triplicate using the DNA extracted from each sediment sample.

### 454 pyrosequencing analysis

To analyze the composition and diversity of the fungal communities in three reservoirs, we used Roche 454 GS FLX pyrosequencing technique. The nuclear ribosomal internal transcribed spacer (ITS) region has recently been used as the standard marker for fungal DNA barcoding, and more ITS sequences are deposited in several databases, giving a large reference for identification of fungal taxa [41-43]. Therefore, ITS region was determined in the present study. The following primers were used: ITS1F (5′-454adapterA-MID-CTTGGTCATTTAGAGGAAGTAA-3′) and ITS4R (5′-454adapterB-TCCTCCGCTTATTGATATGC-3′) (amplifying both ITS1 and ITS2 introns) [42]. Adapter A and Adapter B represented 5′-GCCTCCCTCGCGCCATCAG-3′ and 5′-GCCTTGCCAGCCCGCTCAG-3′, respectively. MID was designed for the barcoding key. ITS1F and ITS4R are fungal-specific primers that correspond to the ITS1 and ITS2 regions, respectively [44].

Each PCR reaction contained 2.5 μl of 10 × reaction buffer, 2 μl of dNTPs (2.5 mM), 1 μl of DNA (20 ng/μl), 1 μl of primer ITS1F (10 μM), 1 μl of primer ITS4R (10 μM), and 0.125 μl of *Taq* DNA polymerase (5U/μl) (TaKaRa, Japan). PCR was carried out in a C1000 Thermal Cycler Gradient (Bio-Rad, USA) with the following cycling protocol: 4 min at 94°C; 34 cycles of 30 s at 94°C, 45 s at 47°C, and 60 s at 72°C; and a final extension for 7 min at 72°C. Samples were then maintained at 4°C until analysis. The resultant PCR products, which were 600 ~ 800 bp fragments of the fungal ITS region, were obtained and purified using a DP209® DNA purification kit (Tiangen Biotechnology Co., Ltd, Beijing, China) following the manufacturer’s recommendations. Small fragments were removed using beads (Beckman Coulter, Brea, CA). Quality and quantity were checked using the Bioanalyzer 2100 (Agilent, Santa Clara, CA, USA) and the Qubit® Fluorometer (Invitrogen, Carlsbad, CA, USA). Emulsion PCR (emPCR) was carried out using a GS FLX emPCR amplicon kit in accordance with streamlined protocols (454 Life Sciences/Roche Applied Biosystems, Nutley, NJ, USA). The ITS regions were sequenced using the Roche GS FLX 454 pyrosequencing platform (Roche Applied Science, USA) by the Shanghai Personal Biotechnology Co., Ltd, China.

After sequencing, the raw sequences obtained were processed using the Quantitative Insights Into Microbial Ecology (QIIME) toolkit [45]. The standard primer sets and barcodes were excluded. Sequence with a quality score lower than 25 was trimmed. Sequences with lengths of less than 200 bp or containing any unresolved nucleotides were removed [5]. Pyrosequencing data were denoising and chimeras were identified and removed from the datasets. To identify potential chimeric sequences, Mothur was used. After removing lower quality sequences, all good-quality sequences obtained by pyrosequencing were clustered into operational taxanomic units (OTUs) with 0.97 cut-off settings. The taxonomic statuses of the tested sequences were classified using the Ribosomal Database Project (RDP) classifier and NCBI Taxonomy Browser [18,21].

### Nucleotide sequence accession number

454 GS FLX pyrosequencing data have been deposited in the National Center for Biotechnology Information-Short Reads Archive (NCBI-SRA) under the accession number SRP 033487.

### Data analysis

The sediment fungal abundance data were analyzed by one-way ANOVA with the Tukey-Kramer honest significant difference test (*P* < 0.01). For bioinformatics analysis, *Chao*1 diversity, abundance-based coverage estimators (ace), and Shannon’s (*H*) and Simpson (*D*) diversity indices were calculated by MOTHUR (http://www.mothur.org/) [5,18,26]. The parsimony test was used to determine the relatedness of the dominant fungal phyla between two reservoirs. Based on the pyrosequencing data, principle component analysis (PCA) was employed to reveal the relationships between the sediment samples and fungal genus data derived from 454 pyrosequencing using SPSS software (Version 16.0, Systat Software, Inc., Chicago, IL, USA). Heat maps and rank-abundance curves were constructed with the *R* statistics software package (Version 3.0.2, USA). The fungal cytoscape network clustering diagram was generated by Cytoscape (Version 2.8.0, USA). The size of each circle indicates the OTU abundance, and the line color indicates the presence of the OTU in this sample. The PCA diagram was built with SigmaPlot for Windows (Version 12.0, Systat Software, Inc., Chicago, IL, USA).
